# Disease aggravation following surgery in a rare patient suspected to Fibrodysplasia (Myositis) ossificans progressiva: a case report

**DOI:** 10.1186/s13256-023-04253-w

**Published:** 2023-12-04

**Authors:** Amir Zarei, Foad Rahimi, Mehryar Khadem, Mansour Moradi, Khaled Rahmani

**Affiliations:** 1 Department of Orthopedic Surgery, Faculty of Medicine, Kurdistan University of Medical Sciences, Sanandaj, Iran; 2 Liver and Digestive Research Center, Research Institute for Health Development, Kurdistan University of Medical Sciences, Sanandaj, Iran

**Keywords:** Fibrodysplasia ossificans progressiva, Myositis ossificans progressiva, Ectopic ossification, Hypoplastic hallux valgus

## Abstract

**Background:**

Fibrodysplasia ossificans progressiva (FOP) as a rare and heritable disorder with the infrequent genetic transmission of the condition is a catastrophic disorder of heterotopic ossification (HO) and a cause of extraskeletal bone formation in humans. Given the lack of effective treatment for this disease, the important point is to avoid aggravating factors such as bone biopsy, surgery, and intramuscular injection.

**Case presentation:**

In this report, we present a 52-year-old female patient, Kurdish ethnic, suspected to FOP who had a surgical intervention on the second toe of the right foot, which subsequently, it caused further deterioration of the disease in the person including necrosis and amputation of the distal phalanx of the second toe.

**Conclusions:**

Although, based on our investigation and the available scientific evidence, surgery may a cause for faster progression and worsening of the FOP disorder, but its proof requires further studies.

## Introduction

Fibrodysplasia ossificans progressiva (FOP), as a rare, heritable and severely disabling genetic condition of connective tissue and progressive heterotopic ossification (HO), is the most catastrophic disorder of HO and a cause of extraskeletal bone formation in humans [1]. According to the literature, the worldwide prevalence of this disorder is approximately 1/2,000,000. There is no difference in the FOP distribution based on ethnic, racial, gender, or geographic factors [2]. The FOP disease was described for the first time in 1692 and so far, more than 600 cases have been reported. Genetically, it is an autosomal dominant disease with an its specific clinical manifestations [1].

FOP is characterized by abnormal ossification in the soft tissue and diffused throughout the body and bilateral Hallux valgus (valgus deviation of the big toe). The onset of the disease is in early childhood and it progressively affects the skeletal system, causing joint dysfunction and immobility. So far, there is no effective treatment for this disease, but the important point is to avoid aggravating factors such as bone biopsy, surgery, and intramuscular injection. The main reason for this disease is the occurrence of mutations in the activin receptor type-1 (ACVR1) gene, which is a member of a protein family called bone morphogenetic protein (BMP) type I receptors as well as it is assessed in the panel of muscle diseases of the Mendel laboratory [3]. Although some cases of fibrodysplasia ossificans especially cases observed after trauma causes involvement of limited organs, but the progressive form of the disease such as the case presented in this report, can be seen in all parts of the body. Due to the rarity of the disease, in this report, we describe a case of a 52-year-old woman, a resident of Sanandaj city, the center for the Kurdistan province in the west of Iran, who was suspected of having FOP, that that the surgery did not lead to her recovery, and she developed more complications and movement restrictions over time.

## Case report

A 52-year-old Kurdish female patient who had been referred about 20 years ago with lesions on her toes, which gradually involved the fingers and then the knee joints on both sides and also the hip joints on both sides. The patient had no family history of this disease and had an IQ appropriate for his age. A surgical intervention had been performed on the second toe of the right foot, which consequently caused necrosis and amputation of the distal phalanx of the second toe, and the wrist joints on both sides are also involved.

**Fig. 1:**
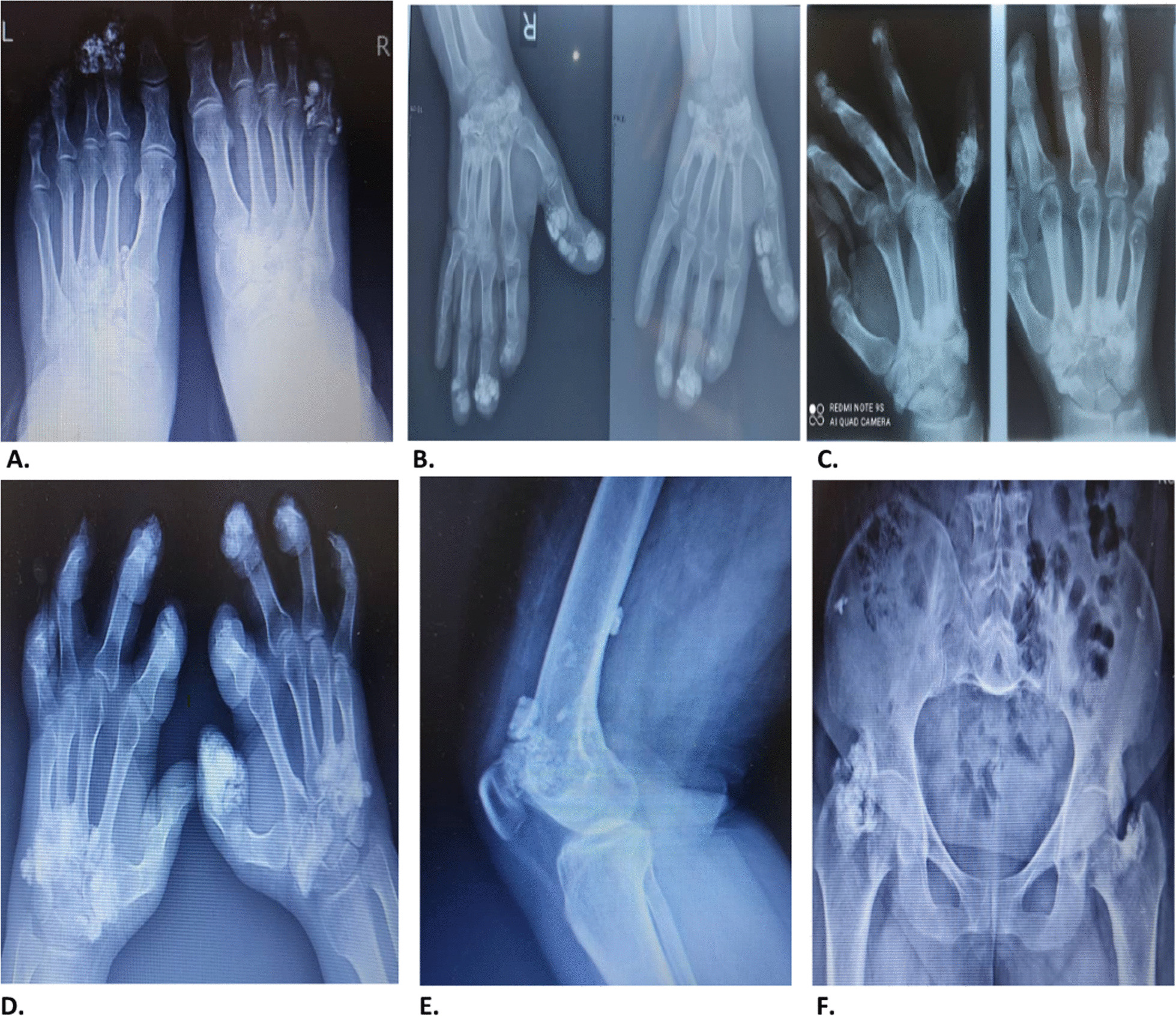
Radiographic features of the study patient. **A** Interphlangeal joints ossification, **B**–**D** Interaarticular ossification in interphlangeal and wrist joints; **E** periarticular ossification of knee joint. **F** Interaarticular and periarticular ossification in hip joints

The first visit of this patient was 20 years ago, at the age of 32, and was due to the involvement of interphalangeal joints of the 3rd and 4th fingers of the right foot. Gradually, the interphalangeal joints of the left foot became involved, and about three years after the first presentation, the interphalangeal joints of the right hand and then the right wrist joint became involved. Shortly after the right hand, signs of disease and involvement of the interphalangeal joints and the wrist joint of the left hand were also observed. After about 10 years, at the age of 42, due to calcium deposition, the right knee and right hip joints were involved, and during the next three years, at by age 45, the patient's joint and tissue around the left knee and left hip joints were also affected. (Fig. 1).

The patient is currently experiencing severe pain and limitation in the joints of their fingers, toes, wrists, knees, and hips bilaterally. The patient’s serum levels of phosphorus, calcium, creatinine, uric acid, and alkaline phosphatase (ALP) were within the normal range in the biochemical tests performed. Laboratory findings of the patient’s urine analysis were negative for protein, glucose, ketone, bilirubin and urobilinogen excretion. The radiographic features of the study patient are pictured in Fig. 1. Since there is no effective treatment for FOP and surgery could not be an option for removing the excess bones, we only used symptomatic medication to help relieve symptoms of FOP, such as pain and inflammation.

It should be noted that the primary draft report was evaluated and approved by the ethics committee of Kurdistan University of Medical Sciences (Ethic code: IR.MUK.REC.1402.054). Furthermore, written informed consent was obtained from the patient before the data collection and intending to the report of her disease information as a case report.

## Discussion

FOP is the most disabling condition of ectopic skeletogenesis [4]. The phenotype of FOP consists of two defining features: congenital malformation of the great toes and progressive HO in characteristic anatomic patterns [5]. Although clinical characteristics of the case presented in our report is partly consistent with FOP, but we could not rule out other disease. FOP is commonly misdiagnosed as aggressive juvenile fibromatosis, soft tissue sarcoma, or lymphedema. Other differential diagnosis include lymphoma, desmoids tumors, brachydactyly (a genetic condition that causes fingers and toes to be shorter than normal), isolated congenital malformations, and juvenile bunions [6].

FOP disease usually starts as swelling of soft tissue with pain and fever, followed by palpable stiffness and misplaced bone formation [1]. The patient under our study exhibits a similar pattern.

Laboratory findings of patients with FOP, including phosphorus and calcium levels, are usually normal, but alkaline phosphatase may increase in acute phases [1]. Laboratory findings of patients with FOP, including phosphorus and calcium levels, are usually normal, but alkaline phosphatase may increase in acute phases [1]. All of these tests were normal for our patient.

Based on existing evidence, the median lifespan age of a FOP patient is approximately 40 years and most patients are wheelchair-bound during the second decade of life and usually die of complications of thoracic insufficiency syndrome [3], while the age of our reported case was 52 and in recent years, her physical condition has worsened.

The use of corticosteroids as first-line treatment at the beginning of disease flare-ups can help reduce intense inflammation and tissue edema. However, undergoing surgery to remove heterotopic bone can trigger explosive new bone growth, and we observed disease aggravation after surgery attempts in our case [7]. Previous evidence and this study's results suggest that surgical attempts are not strongly recommended.

In general, the mutation in a specific ACVR1/ALK2 gene may be a necessary cause of HO in FOP, it is not sufficient cause for inducing flare-ups of the disease that resulting in progressive disability. Determining the role of the immune system, the responses of resident progenitor cells, and the biochemical aberrations of the soft tissue microenvironment that can involve in the induction of flare-ups of the disease requires further studies.

Finally, it can be concluded that the initial surgery on the toe could not stop the progress of the disease. Based on the history and referrals of the patient, even the disease had a progressive course following the surgery. However, according to this evidence, it cannot be definitively concluded that surgery was or was not a risk factor for worsening the patient's condition.

## Conclusion

In this report we presented a suspected FOP case which had progressively ossification in different joints of the body following a surgery on her toes. Although, based on the investigation of this case and the available scientific evidence, surgery may be a cause for faster progression and worsening of the FOP disorder, but its proof requires further studies.

## Data Availability

Please contact the corresponding author for data requests.

## Abbreviations

FOP: Fibrodysplasia ossificans progressiva
HO: Heterotopic ossification
ACVR1: Activin receptor type-1
BMP: Bone morphogenetic protein

## References

[1] Pignolo RJ, Shore EM, Kaplan F (2011). Fibrodysplasia ossificans progressiva: clinical and genetic aspects. Orphanet J Rare Dis.

[2] Shore EM (2005). The genetics of fibrodysplasia ossificans progressiva. Clin Rev Bone Mineral Metabo.

[3] Kaplan FS (2008). Fibrodysplasia ossificans progressiva. Best Pract Res Clin Rheumatol.

[4] Pignolo RJ, Shore EM, Kaplan FS (2013). Fibrodysplasia ossificans progressiva: diagnosis, management, and therapeutic horizons. Pediatric Endocrinol Rev.

[5] Kaplan FS (2005). The phenotype of fibrodysplasia ossificans progressiva. Clin Rev Bone Mineral Metabol.

[6] Kitterman JA (2005). Iatrogenic harm caused by diagnostic errors in fibrodysplasia ossificans progressiva. Pediatrics.

[7] Hair MS, Peeper JL, Metabolism M (2005). The international fibrodysplasia ossificans progressiva association. Clin Rev Bone Mineral Metabol.

